# Editorial: Genomic insights into sheep and goat breeding efficiency

**DOI:** 10.3389/fvets.2025.1717356

**Published:** 2025-11-04

**Authors:** Fei Hao, Mingli Peng, Ziyang Xu, Xiao Zhang, Jiawei Wang, Rui Ding

**Affiliations:** ^1^Key Laboratory of Reproductive Regulation and Breeding of Grassland Livestock, School of Life Sciences, Inner Mongolia University, Hohhot, China; ^2^Northumbria University, Newcastle upon Tyne, United Kingdom,

**Keywords:** Grassland Livestock, genomic, gene, phenotype, breeding efficiency

Grassland Livestock, as the gene, phenotype, Breeding efficiency and ecosystem, provide high-quality livestock products, such as meat, milk and down, that are essential for human survival, maintain the energy flow between forage and humans, and also carry the historical connection between thousands of years of nomadic civilization and the transformation of modern animal husbandry (1–4). With the development of cell biology, molecular biology, and genomics, researchers have conducted extensive studies on the periodic growth of cashmere goat hair follicles, reproductive efficiency of sheep, and improvements in dairy cow production (5, 6). This scaffolds a resolution to the dichotomy between “protecting grassland ecology” and “improving livestock productivity.” Such related research is a core focus in animal husbandry, veterinary science, genetics, and ecology (7).

In recent years, research on grazing livestock has progressed from traditional phenotypic observations and population surveys to a new stage of elucidating the molecular mechanisms of phenotypes and molecularly-informed breeding (8). In-depth research has been conducted on key economic traits, such as growth, reproduction, disease prevention, and the control of grazing livestock (9). Related research provides scientific solutions to the problems faced in grassland animal husbandry, such as long breeding cycles, difficult trait improvements, and insufficient ecological adaptability (10).

Consequently, *Frontiers in Veterinary Science* has established a Research Topic, “Genomic Insights into Sheep and Goat Breeding Efficiency,” carefully selecting 13 high-quality, original research articles focusing on the field of grazing livestock. These involve important grazing animals such as sheep, goats, yaks, Yunling cattle, and donkeys, and cover the complete research chain from “genetic resource evaluation to functional gene mining to production trait regulation to molecular breeding application.” These papers cover the molecular basis of important biological characteristics of grazing livestock from different perspectives, providing references for promoting innovation in grazing livestock scientific research and promoting sustainable commercial development.

In terms of genetic resources and adaptive evolutionary research in grazing livestock, multiple studies have focused on the genomic characteristics of typical livestock species, providing a molecular basis for the conservation of genetic diversity and stress-resistant breeding. Tang et al. used 10X whole-genome sequencing to conduct single nucleotide polymorphism analysis on seven local horse breeds in Xinjiang, China, and found high population genetic diversity among these local breeds. Marked genetic differences from other horse breeds from Europe, central Asia, western Asia, and China were reported, elucidating differences in distribution patterns, evolutionary characteristics, and genetic diversity.

Zhang S. et al. conducted whole-genome resequencing of six yak populations in south-western China and found rich genetic diversity in yaks from this region. Tibetan yaks showed lower nucleotide diversity because of geographical isolation, whereas Muli yaks were substantially different from the other groups. Strong candidate genes related to high-altitude adaptation, growth, and development were found, addressing a gap in genomic research on yak populations in south-western China.

Dang et al. successfully constructed a genomic copy number variation (CNV) map for Yunling cattle that will facilitate an in-depth analysis of the genetic mechanisms underlying the formation of economic traits, such as subcutaneous fat thickness and *longissimus dorsi* muscle area in Yunling cattle.

By comparing selective sweep signals between Iranian domestic sheep and wild Mouflon sheep, Taheri et al. found that genes, such as *ADGRB3* and *CAPN2*, in domestic sheep were strongly correlated with economic traits, such as body weight and milk yield, whereas genes such as *ACAN* and *MGST3* in wild sheep were related to adaptive traits, such as daily weight gain and bone weight. This indicates the differential effects of artificial and natural selection on the sheep genome, providing a new perspective for the utilization of sheep genetic resources.

Another core focus of this Research Topic is the regulatory molecular mechanisms of important production traits. Multiple papers in this Research Topic target key traits, such as hair follicle development, reproductive performance, growth, and meat quality, mining many functional genes and regulatory pathways with potential application in the field. In hair follicle development research, Han et al. used proteomics techniques to analyze differences between the telogen and anagen phases of secondary hair follicles in cashmere goats. They found that ADAM17, SFRP1, and PPP1CA proteins might promote hair follicle cycle transition by regulating signaling pathways, such as Notch and Wnt. Zhang C. et al. used multi-omics joint analysis techniques to elucidate the important role of ribosomal proteins during the hair follicle cycle transition. Yuan et al., focusing on the depilation characteristics of Dorper sheep, screened hair follicle development-related genes, such as *DBI, FZD3*, and *ZDHHC21*, providing new targets for the regulation of the sheep hair follicle cycle. Related research provides references for studying the mechanisms of hair follicle growth and development in goats and sheep.

In the field of reproductive efficiency traits, Quan et al. systematically analyzed the reproductive performance of Huanghuai goats and found an average litter size of 2.74 and an annual reproductive rate of 418.96%. Combined with transcriptome sequencing technology, they screened candidate genes, such as *PTX3* and *MMP13*, for prolificacy traits.

In the field of meat yield and meat quality-related traits, Liu et al. genotyped non-synonymous single nucleotide polymorphisms of three candidate genes (*KIAA1217, SNTA1*, and *LTBP1*) in Ujimqin sheep and performed association analysis between the genotyping results and growth traits. These results provide important data for genetic marker-assisted selection of Ujimqin sheep.

Peng et al., using transcriptome data from the longissimus dorsi muscle of Guangling donkeys, analyzed the importance of long non-coding RNAs in intramuscular fat deposition, expanding the research dimension of non-coding RNAs in the regulation of grazing livestock traits.

Research in molecular breeding technology innovation and application has focused on addressing the bottlenecks in breeding in small populations and optimizing trait prediction models to provide new methods for improving the breeding efficiency of grazing livestock. Zhang S. et al. analyzed the genetic structure of 485 Xinjiang Brown cattle and 2,633 Chinese Holstein cattle to establish a cross-bred joint reference population. They also evaluated the estimated genomic breeding values of milk production traits in Xinjiang Brown cattle. This study provides basic data for genomic prediction and selection in dairy cattle. Qi et al. screened candidate genes related to tail-type using whole-genome re-sequencing in Mongolian (short, fat-tailed) and Bamei (long, thin-tailed) mutton sheep. Of these, *PDGFD, GLIS1*, and *VRTN* were strongly associated with tail-fat deposition and tail length. This study provides a new theoretical basis for the molecular breeding of tail-type traits in sheep.

Li et al. conducted transcriptome sequencing of the hypothalamus of Jining Gray goats, identifying 237 differentially-expressed, long non-coding RNAs and analyzed their regulatory role in goat sexual maturity, providing a reference for improving goat reproductive efficiency.

In summary, the 13 research reports in this Research Topic comprehensively present the current research frontiers in the field of grazing livestock, including an in-depth analysis of the genetic resources of livestock species, such as yaks and Yunling cattle; innovative exploration of the regulatory mechanisms of key traits, such as hair follicles and reproduction; and practical breakthroughs in technologies, such as cross-breeding. These achievements not only enrich the theoretical system of grazing livestock biology but also provide scientific support for the synergistic advancement of animal health, ecological protection, and commercial development under the “One Health” concept.
